# Suitability of Commercial Transport Media for Biological Pathogens under Nonideal Conditions

**DOI:** 10.1155/2011/463096

**Published:** 2011-10-30

**Authors:** Kyle Hubbard, Gregory Pellar, Peter Emanuel

**Affiliations:** ^1^Excet, Inc., 8001 Braddock Rd Suite 303, Springfield, VA 22151, USA; ^2^Edgewood Chemical Biological Center, U.S. Army, RDECOM, Edgewood Area, Aberdeen Proving Ground, MD 21010, USA; ^3^Noblis, Inc., 3150 Fairview Park Drive, Falls Church, VA 22042, USA

## Abstract

There is extensive data to support the use of commercial transport media as a stabilizer for known clinical samples; however, there is little information to support their use outside of controlled conditions specified by the manufacturer. Furthermore, there is no data to determine the suitability of said media for biological pathogens, specifically those of interest to the US military. This study evaluates commercial off-the-shelf (COTS) transport media based on sample recovery, viability, and quality of nucleic acids and peptides for nonpathogenic strains of *Bacillus anthracis*, *Yersinia pestis*, and Venezuelan equine encephalitis virus, in addition to ricin toxin. Samples were stored in COTS, PBST, or no media at various temperatures over an extended test period. The results demonstrate that COTS media, although sufficient for the preservation of nucleic acid and proteinaceous material, are not capable of maintaining an accurate representation of biothreat agents at the time of collection.

## 1. Introduction

The anthrax attacks of 2001 highlighted gaps in the US bio-terrorism related preparedness [1]. The large numbers of incoming samples overwhelmed on-site mobile laboratories and required shipping sample off-site for analysis of viability in support of large-scale remediation activities such as those that occurred at the Brentwood Postal Offices and the Hart Senate Office building [2]. On-site sample extracts were stored in water at 4°C or −20°C for longer storage times. For off-site analysis, swabs were shipped dry on ice. Retrospective analysis of the events clearly indicates that a lack of standardized collection and processing techniques for environmental samples complicated contamination and clean-up assessment [1, 3]. Biological remediation activities, longitudinal clinical studies, and verification activities in support of military and treaty exploitation each share a common theme—a requirement to collect samples and preserve them for examination and analysis at a future date. In situations where samples must be analyzed by offsite laboratories, significant time can elapse between when a sample is collected and when it is analyzed. In these situations a biological sample will often lose viability for re-growth or degrade and denature which makes subsequent analysis by PCR or immunoassay more difficult. It is imperative to have a process for short- and long-term storage that is efficient and preserves sample integrity over time. For collection teams that support military or international treaty verification organizations, that need is further complicated by the difficulty in maintaining the cold chain, the highly pathogenic nature of samples that may need to be collected, and in the great distances that a sample may need to be shipped until it can be analyzed by a properly equipped laboratory. The use of commercially available collection and transport media is attractive due to their worldwide availability and low price. 

The following study was conducted to assess the potential for one type of commercially available collection and transport system that was developed for clinical samples and to determine its potential to be adapted for use with four prototypical agents of biological origin that may be of interest to military and treaty verification organizations. Each transport system contained a sterile, rayon-tipped swab applicator used to collect the sample and a tube containing transport medium into which the swab applicator is placed after sampling. We examined the use of these particular COTS transport media for their ability to maintain organism or toxin viability, as well as their suitability for maintaining detectable levels of nucleic acid and protein over a range of environmental conditions. 

The agents chosen for this study included *Bacillus anthracis*, a gram-positive endospore-forming species and the non-spore-forming, gram-negative *Yersinia pestis*. Strains with reduced pathogenicity (*Bacillus anthracis* Sterne and *Yersinia pestis* A1122, resp.) were used for testing. The vegetative form of *B. anthracis* was used because spores do not require liquid transport medium for stabilization. However, the suitability of transport media for preserving vegetative cells has not been determined. In addition, given the expansion in global biosurveillance, the utility of these COTS kits for environmental sampling may not be limited to traditional weapons and spore deposition, but could have applicability for animal carcasses or human patients where the vegetative form would prevail. The ricin holotoxin purified from defatted castor beans was the protein toxin bio-threat agent tested. Venezuelan equine encephalitis (VEE) virus was chosen as an example of an enveloped and single-stranded RNA virus as well, being associated with human disease. The TC-83 vaccine strain of VEE was chosen for testing due to its lower pathogenicity.

## 2. Materials and Methods

### 2.1. Biological Agents, Mediums, and Growth Conditions

All chemicals were molecular grade and purchased from Fisher Scientific (Suwanee, Ga, USA). *Bacillus anthracis* (Sterne strain) and *Yersinia pestis* A1122 (YPA1122) were obtained from the Critical Reagents Program (CRP) (Aberdeen Proving Ground, Md, USA). The Sterne strain was streaked out on TSA plates (Becton Dickinson Inc. Franklin Lakes, NJ), and colonies appeared following incubation at 37°C for 24 hours. A single colony was added to 5 mL of sterile TSB (Remel Inc., Lenexa, KS) and allowed to grow at 37°C with rotary aeration at 180 rpm for 24 hours. The culture was expanded by taking 1 mL from the starter culture and adding it to 500 mL sterile TSB. The culture was allowed to grow at 37°C at 180 rpm for 16–24 hours. Growth of YPA1122 followed a similar course with the exceptions that the cultures were incubated at 30°C for 42–48 hours. Venezuelan equine encephalitis TC-83 virus was obtained from the Critical Reagents Program (Aberdeen Proving Ground, Md, USA). The frozen stock had a concentration of 4 × 10^7^ pfu/mL in cell-freezing medium (catalog number 12648-010, Invitrogen Inc., Carlsbad, Calif, USA). Cell culture supplies were obtained from VWR international (West Chester, Pa, USA). Virus titer was determined by plaque assay in a Vero cell line obtained from the American Type Culture Collection (ATCC, catalog number CCL-81, Manassas, Va, USA). Each well of a six-well microtiter plate was loaded with 5 × 10^5^ Vero cells (2.5 × 10^5^/mL) the previous day and allowed to incubate overnight at 37°C with 5% CO_2_ and 100% humidity before the virus was introduced. After the one hour incubation at 37°C with mild shaking, the virus was removed and 2 mL of a 1 : 1 mixture of 2× Modified Eagles Medium (MEM) (catalog number 11935-046, Invitrogen, Carlsbad, Calif, USA) and 2% seaPlaque agarose (catalog number 12001-898, Cambrex, Charles City, Iowa, USA) were overlaid. The mixture was left at room temperature until solid, followed by incubation for 48 hours at 37°C 5% with CO_2_ and 100% humidity. Ricinus communis agglutinin II (RCA 60, Ricin), 5 mg/mL in 10 mM phosphate, 150 mM NaCl, pH 7.8 with 0.08% sodium azide as a preservative was purchased from Vector Laboratories (Burlingame, Calif, USA). The ricin was stored at 4°C when not in use.

### 2.2. Swab Preparation

The bacterial cultures were centrifuged at 2200 rcf for ten minutes at 10°C in order to pellet cells. The cell pellets were then washed in 10 mL phosphate buffered saline, 14 mM NaCl, 0.3 mM KCl, 1 mM Na_2_HPO_4_, 2 mM KH_2_PO_4_ (pH 7.4) w/0.1% Triton X-100 (PBST) before being resuspended in a small volume of PBST to generate a bacterial loading stock. In order to get a preliminary determination of concentration, the bacterial stock was diluted in PBS and optical density was determined using a Spec 20D+ spectrophotometer (Thermo Fisher Scientific Inc., Waltham, Mass, USA) at a wavelength of 600 nm and compared to a standard curve derived from known bacterial concentrations. The stock was then diluted in PBST in accordance with spectrophotometer data to obtain a concentration of approximately 3 × 10^8^ cfu/mL and 1.6 × 10^9^ cfu/mL for *B. anthracis* Sterne and YPA1122, respectively. 100 *μ*L of the stock was then absorbed onto a rayon swab which was then placed into a tube containing transport media. In addition to testing the following media: PBST, liquid Amies (catalog number 140C), and liquid Stuart (catalog number 141C), swabs were also placed into dry tubes (catalog number 155C). All transport media were purchased from Copan Diagnostics Inc. (Corona, Calif, USA), except for PBST, which was prepared on site in sterile empty transport tubes. Swabs were tested in duplicate for each time point and temperature. Control swabs for each time point were loaded with 100 *μ*L PBST, but were incubated only at 25°C. 

Two ricin stocks were created by diluting with sterile-filtered distilled water to a final concentration of 5 *μ*g/mL for the high spike experiment (HS), and 50 ng/mL for the low spike (LS) experiment. Rayon swabs were spiked with 100 *μ*L of the high concentration or low concentration, thereby loading 500 ng and 5 ng ricin per swab, respectively. Three transport conditions were examined: sterile water, dryness, and protein transport medium (PTM). PTM is made up of 0.1% BSA and 1.0% Triton X-100 in sterile water and was made in-house. For the liquid transport media, one milliliter of each was added to sterile transport tubes containing a sponge (kindly provided by Copan Diagnostics Brescia, Italy). 

The VEE TC-83 frozen stock was diluted 1 : 20 using Earl's modified eagle's medium (EMEM), catalog number 30-2003 (ATCC, Manassas, Va, USA). 100 *μ*L of the 2 × 10^6^ pfu/mL loading stock was absorbed onto rayon swabs which were then cut off into plastic tubes containing 3 mL M4RT (Remel, catalog number R12505) or UTM (Copan, catalog number 402C) viral transport media as well as PBST and tubes containing no transport medium (dry). Each tube also had three 1mm glass beads to aid in extraction. In addition, one control swab for each transport medium was loaded with 100 *μ*L PBST.

### 2.3. Viability Testing

Swabs spiked with *Bacillus anthracis* Sterne and *Yersinia pestis* A1122 were stored at one of four temperatures (−70°C, 4°C, 25°C, or 45°C) for up to 60 days. Two swabs were removed per time point and the swab heads extracted by vortexing on a large area mixer (70% maximum pulse, for two minutes) in 5 mL PBST. For day 0, extractions and swabs were processed within one hour of being loaded so as to avoid irreversible binding to swab heads. 100 *μ*L of the extract was serially diluted in PBST and plated onto tryptic soy agar plates in triplicate. In addition to quantifying the viable titer associated with the swab, the residual transport media was also serially diluted in PBST to determine if viable organisms diffused from the rayon into the transport media. *Bacillus anthracis* Sterne *was* incubated for 16–24 hours at 37°C, while *Yersinia pestis* A1122 was incubated for 42–48 hours at 30°C, after which colonies were counted using the Q-Count colony counter (Advanced Instruments Inc, Norwood, Mass, USA) to determine bacterial concentration. 

Swabs spiked with VEE virus were stored at three temperatures (−70°C, 4°C, or 25°C) for up to 21 days. Virus was extracted directly from the transport tube by vortexing for two minutes (dry swabs were extracted in 3 mL sterile deionized water). 100 *μ*L transport medium was serially diluted in 900 *μ*L EMEM. 500 *μ*L of each dilution was added to the Vero cells as described above to determine viral titer.

### 2.4. ECL Immunochemistry

The protein signature of the bacteria, virus, and toxin was determined using the BioVeris Inc M1M electrochemiluminescence (ECL) system (Gaithersburg, M, USA). ECL minitube immunoassays were purchased from the Critical Reagents Program (CRP) (Aberdeen Proving Grounds, Md, USA). The assays use two antibodies specific for the antigen of interest in a single-tube lyophilized reagent format. In addition, positive controls include a low-quantity (PCL) and a moderate-quantity (PCM) of positive control protein. This allows each of the stored samples to be semiquantitatively analyzed in comparison to the positive controls. Swab head extract (100 *μ*L) was added directly to the lyophilized reagents. The M1M is completely automated and performs all incubations and reagent additions according to a preprogrammed methodology for each assay. *B. anthracis* Sterne, *Y. pestis* A1122, ricin, and VEE extracts were all assayed using ECL MINItubes available from the CRP.

### 2.5. Real-Time PCR

Real-time PCR was utilized for nucleic acid detection. *B. anthracis* Sterne and *Y. pestis* A1122 were assayed on the Roche LightCycler 2.0 using the Idaho Technologies (Salt Lake City, Utah, USA)* B. anthracis* Sterne Target 1 kit (catalog number 3828) and *Y. pestis* A1122 Target 1 kit (catalog number 3831), respectively. The real-time PCR assay for the test samples consisted of 20 *μ*L of the swab head extract and 20 *μ*L of kit reconstitution buffer added to the lyophilized reagents. 

Viral nucleic acid detection was achieved using the Idaho Technologies JBAIDS Kit catalog number JRPD-ASY-0114. All real-time PCR assays were used according to manufacturers' recommendations. The viral swab extracted was diluted 10-fold with water, and 40 *μ*L of the diluted sample was added to reconstitute the lyophilized real-time PCR reagents. Crossing points were determined using the LightCycler 4.0 software algorithm (Roche Diagnostics, Indianapolis, Ind, USA).

## 3. Results

### 3.1. Effect of Nonideal Storage Conditions on Biothreat Agent Viability

All data is expressed as percentage recovery relative to the total specimen loaded onto the swab head on day 0. In order to capture and quantify the total amount of viable organism present at a specific time point, the values representing the total of the extractions from the swab heads and from the residual transport media are shown in Table 1.

#### 3.1.1. *Bacillus anthracis* Sterne

Temperature appeared to exert the greatest influence on the viability of *B. anthracis* Sterne. The highest recovery rates were measured for samples stored at 25°C in liquid Amies, liquid Stuart, and PBST (Table 1). Viability was also observed under dry storage conditions at that temperature. Storage at 4°C and 45°C resulted in the greatest loss of viability for all media tested by day 60 (Table 1). Swabs stored at −70°C did result in an initial decrease in viability of greater than 96% for all transport media; however, stronger long-term viability was observed over the 60-day test period than storage at either 4°C or 45°C (Table 1). *B. anthracis* stored at 25°C in both liquid Amies and liquid Stuart resulted in an increase in mean recovery starting on day 3 (Table 1). The samples were visualized using phase-contrast microscopy (see supplemental data available online at doi:10.1155/2dl/463096), which showed the increase in recovery was the result of sporulation of bacilli chains into individual spores rather than germination. 

#### 3.1.2. *Yersinia pestis * A1122

Like *B. anthracis*, recovery of* Y. pestis *was largely dependent on temperature and storage media. *Y. pestis* viability was best preserved at 4°C in liquid Stuart, displaying a 68% reduction in original load material, while viability in PBST decreased by 97.4% and viability in liquid Amies decreased by 90% after 60 days. All transport media failed to maintain appreciable viability after 60 days at 25°C (Table 1). PBST did not preserve viability as well as Stuart and Amies at 45°C with no viability detected after one day. Stuart and Amies were able to maintain detectable viability for at least 24 hours at 45°C. Swabs stored at −70°C in both liquid Amies and liquid Stuart showed the ability to maintain long-term viability. Freezing did cause an initial decrease in viability before day 1 for all transport media conditions; however, viability remained static in liquid Amies and liquid Stuart and under dry conditions after that during the 60-day test period (Table 1). Dry storage exhibited a compete loss of viability after 14 days at 25°C and a near-complete loss of viability by 28 days at 4°C.

#### 3.1.3. VEE

 Overall, viability was higher when samples were stored at colder temperatures in URM and M4RT (Table 1). No viable virus was recovered from swabs stored in PBST at any temperature. At −70°C, virus survival was aided when stored in UTM and M4RT, exhibiting a higher rate of recovery than samples stored on dry swabs across the 21-day test period (Table 1). The recovery efficiency for UTM and M4RT virus transport media was also pronounced for samples stored at 4°C where there was no viable virus in either PBST or under dry conditions (Table 1). Storage at 30°C was largely inhibitory to recovery of virus. UTM preserved VEE viability for only 7 days while none of the other transport methods were positive for viable virus, although that result is highly variable. 

### 3.2. Nucleic Acid Detection of Bacterial Biothreat Agents Using Real-Time PCR

The Real-Time PCR data was consistent without any significant observable increases or decreases in recoverable genetic material across all storage conditions and time points, independent of biothreat agent tested; therefore, data will be presented as supplemental.

### 3.3. ECL Immunodetection of Bacterial Biothreat Agents

A four plus system was used to indicate the number of tests that were positive according to ECL immunodetection. Duplicate swabs were extracted at each time point and set up into duplicate detection assays. Thus, there were a total of four luminescent readings by which to assess whether a sample was positive (+) or negative (−) for agent detection.

#### 3.3.1. *B. anthracis* Sterne

Protective antigen, the protein detected by this particular ECL kit, was universally detected at −70°C, but not detected at all at 45°C, regardless of the presence of transport media or the storage temperature (Table 2). A positive signal was detected on day 1 at 4°C for all storage conditions but was absent by day 7, except under dry swab conditions which had detectable amounts of protective antigen throughout the course of the study. In addition, only PBST and liquid Stuart showed any positive protein detection at 25°C and only on day 1 (Table 2). 

#### 3.3.2. *Y. pestis* A1122

 All *Y. pestis* samples were positive over the time course regardless of the storage temperature (Table 2). 

#### 3.3.3. VEE

The ECL results were consistently positive throughout the 21-day time course. VEE viral protein was detectable for all time points and under all storage conditions; this despite the lack of a viable virus in PBST (Tables 1 and 2). 

#### 3.3.4. Ricin

Ricin holotoxin was detected using an ECL-based immunoassay as described above. Using the 500 ng high spike concentration (HS) resulted in a much different relative luminescence signal when compared to the 5 ng low spike concentration (LS) (Table 3). This was true regardless of the absence or presence of transport buffer and at all storage temperatures examined. Protein transport media (PTM) almost universally provided the most robust detection levels (Table 3) at every temperature and time point. However, storage in water also resulted in comparable detection levels to storage in PTM at 25°C (Table 3). As the temperature increased from −70°C to 45°C, the immunodetection of ricin holotoxin decreased (Table 3) in all transport media. Most striking is the fact that ricin protein was detectable from the high spiked swabs throughout the 60-day time course when stored at 45°C in liquid transport media (Table 3).

## 4. Discussion

An exhaustive number of studies have examined the efficacy of various sampling kits and techniques for removing bio-threat agents from a wide range of environmental conditions, while others still explored the efficacy of different transport media on the viability of bacterial samples [4–18]. This study evaluates unmodified commercial off-the-shelf (COTS) transport media based on sample recovery, viability, and quality of nucleic acid and peptides for nonpathogenic strains of *B. anthracis* Sterne, *Y. pestis* A1122, VEE, and ricin over a 60-day period. The results reported here demonstrate that COTS media evaluated here are not capable of maintaining an accurate representation of the quantity of biothreat agent at the time of collection. 

A comparison between *B. anthracis* and *Y. pestis* enumeration over the 60-day test period clearly shows that while total numbers of *B. anthracis* colony counts increase, the *Y. pestis* counts decrease. This difference is likely attributable to how each organism responds to stress in a nutrient-deleted environment [19]. Higher temperatures and the presence of liquid Amies or Stuart transport media, which are both nutrient poor, promoted sporulation [20]. Lower temperatures inhibited *B. anthracis *Sterne sporulation, which in turn resulted in a rapid loss of viability of the vegetative cells. This suggests that long-term storage at elevated temperatures diminishes recovery of viable organisms except for microorganisms capable of sporulation in nutrient-poor transport media. Unfortunately, if a sample slowly converts from the vegetative to the spore state, the sample which is analyzed in the lab is not accurately portraying the sample that was originally collected on the swab at time zero. Downstream forensic lab analyses that require an accurate snapshot of the original sample at time zero will require a transport medium that contains sporulation inhibitors while maintaining vegetative cell viability.

The ECL protein detection assay for *Y. pestis* A1122 was more robust than the same assay for *B. anthracis *Sterne. Using the *Y.-pestis*-antibody-based ECL assay, it was possible to detect protein for weeks after loss of viability. This is vividly apparent for *Y. pestis* A1122 spiked samples stored at 45°C. In contrast, *B. anthracis* protective antigen were only detected for a short period of time from samples stored at −70°C. The differences observed in protein signature detection between *Y. pestis* A1122 and *B. anthracis *Sterne was also likely due to the latter's ability to sporulate. Protective antigen will not be as widely expressed in *B. anthracis* in a sporulative state, as general transcription and translation become dormant [21–23]. If protein signature detection using ECL was performed on spore coat proteins on day 60, the results would likely look markedly different from those observed in this study using protective antigen and needs to be addressed in future studies.

The results from the real-time PCR assay were similar to those seen with the ECL assay; there was no correlation with viability. There were detectable levels of nucleic acid for all transport media at every temperature over the 60-day test period for both *Y. pestis* and *B. anthracis*. 

Storage of VEE virus in viral transport media is required if any viability is to be maintained after seven days unless the sample is frozen. Even for frozen storage, the commercial viral transport media M4RT and UTM provided significantly better recovery than storage in PBST or under dry conditions. The rapid loss in VEE virus viability when stored in PBST is likely due to the disruption of the viral envelope by the presence of detergent [24]. 

Viral protein stability was not enhanced by either M4RT or UTM; however, dry storage did reduce viral protein recovery when not stored frozen. No definitive conclusions can be drawn for the viral nucleic acid signature stability from the data collected, except that protein was detectable for all temperature and storage conditions during the 21-day test. 

Reverse-transcription real-time PCR specific for VEE revealed detectable amounts of nucleic acid for all transport media at every temperature over the 21-day test period. This was in stark contrast to the viability results, demonstrating live organism is not absolutely necessary for nucleic acid detection. 

This series of experiments demonstrates that VEE viability is more sensitive to storage conditions than detection of the molecular targets using ECL or reverse-transcription real time PCR assays. Viability studies should be expanded to determine more precisely how long VEE can be recovered from samples stored at 4°C in transport media since by day 21, very little drop in titer was recorded. Additional time points should also be examined for samples stored at 30°C to more precisely determine the rate of virus decay at this temperature in virus transport media. The time course studies should also be extended for the ECL and real-time PCR assays. 

Storing ricin-contaminated swabs in PTM or water while refrigerated or frozen resulted in higher protein detection than dry swabs under the same conditions at the completion of the 60-day test period. However, due to the limited number of samples and semiqualitative nature of the assay, further testing and ricin activity assays would be required to determine if a significant difference exists between sterile water and PTM in protein signature stability. Frozen storage at −70°C provided the best stability as evidenced by the consistent detection of the lightly contaminated swabs. For some of the storage conditions, there was ricin present at day 60 after it appeared to have been undetectable by day 28, although this is likely an artifact of the experimental design. As ricin has no reproductive capabilities, the increase in detectable material at day 60 is likely due to the evaporation of the buffer used to spike the ricin onto the swab artificially increasing the concentration, although more data between days 28 and 60 would be needed to support this claim. Furthermore, because of the ECL in an immunological assay, there is no way to tell if the ricin detected at day 60 was active.

This study evaluated the use of common COTS transport and stabilization swab systems for various biothreat agents. The results demonstrate that these type of COTS transport systems, typically used in clinical settings, can be useful in storage and transport of biothreat agents in environmental settings where conditions may be less than ideal only if obtaining a “yes” or “no” answer is sufficient and sensitive molecular assays are available. The viability studies conducted suggest that the types of COTS transport and stabilization tested are not capable of maintaining an accurate representation of the biothreat agent at the time of collection and should not be used for forensic analyses. This is especially true for spore-forming bacteria. Future transport media design will need to address this issue.

## Supplementary Material

Supplemental Figure 1 depicts microscopic images, captured using a 100X objective, of*B. anthracis* sporulation in PBST, liquid Amies and liquid Stuart transport media on day seven. Vegetative cells are seen as long filaments in the stock culture on day zero in PBST (panels on the far left). Spores are observed as small refractive bodies either within the filamentous cells (or cell fragment) or free within the transport media (panels on the right). Supplemental Figures 2–4 demonstrate nucleic acid detection using real-time PCR for *Y. pestis*, *B. anthracis* and VEE respectively. Each figure summarizes the real-time detection data for each transport media at each storage temperature: −70°C (A), 4°C (B), 25°C (C) and 45°C (D) for *Y. pestis* and *B. anthracis*, −70°C (A), 4°C (B) and 30°C (C) for VEE. Data points represent the average C_t_ value for two PCR reactions over the 60-day test period.

## Figures and Tables

**Table 1:** Viability of *B. anthracis*, *Y. pestis,* and VEE. The numbers shown are percentages of total specimen loaded onto the rayon swab heads on day 0. In an effort to consolidate the large number of samples being extracted, VEE collection did not start until day 7 and persisted only until day 21. Boxes have been grayed out where there is discordance between bacterial and viral collection points. In addition, no data was taken for a sample after it displayed 0% viability for two successive time points, indicated by a (−).

Biothreat agent	Storage temp.	Storage Media	Day 1	Day 3	Day 7	Day 14	Day 21	Day 28	Day 60
*Bacillus anthracis*	−70°C	PBST	3.8 ± 0.3	0.91± 0.13	5.0 ± 0.80	4.73 ± 0.91	1.76 ± 0.36	0.69 ± 0.1	3.48 ± 0.42
		Amies	3.69 ± 0.4	2.55 ± 0.6	7.61 ± 1.6	13.5 ± 3.8	3.1 ± 0.7	4.26 ± 0.5	3.8 ± 0.6
		Stuart	2.52 ± 0.5	2.92 ± 0.5	17.4 ± 3.6	9.83 ± 1.5	5.57 ± 0.7	7.97 ± 1.6	7.47 ± 1.1
		Dry	0.9 ± 0.03	4.52 ± 0.6	2.61 ± 0.2	1.92 ± 0.3	5.72 ± 0.3	1.69 ± 0.1	6.46 ± 0.6
	4°C	PBST	4.7 ± 0.37	0.14 ± 0.03	0.08 ± 0.02	0.01 ± 0.001	0.87 ± 0.001	0.002 ± 0.001	0.005 ± 0.001
		Amies	7.57 ± 0.4	0.85 ± 0.2	0.61 ± 0.1	0.05 ± 0.02	0.006 ± 0.001	0.004 ± 0.001	0.004 ± 0.001
		Stuart	14.2 ± 3.57	2.25 ± 0.4	0.8 ± 0.07	0.3 ± 0.04	0.006 ± 0.001	0.002 ± 0.001	0.003 ± 0.001
		Dry	1.25 ± 0.05	0.9 ± 0.06	0.006 ± 0.001	0.002 ± 0.001	0.002 ± 0.001	0.002 ± 0.001	0.003 ± 0.001
	25°C	PBST	73.1 ± 15.9	52.4 ± 5.94	114 ± 17	62.1 ± 6.32	79.6 ± 3.85	71.3 ± 4.37	56.7 ± 5.23
		Amies	68.1 ± 14.8	102 ± 11.6	451 ± 22	372 ± 46	293 ± 39	298 ± 28	258 ± 11
		Stuart	87.4 ± 2.61	122 ± 6	171 ± 23	162 ± 18	195 ± 28	215 ± 18	249 ± 22
		Dry	7.93 ± 3.18	2.92 ± 0.7	0.8 ± 0.2	0.8 ± 0.1	2.81 ± 0.5	0.4 ± 0.04	0.6 ± 0.2
	45°C	PBST	0.01 ± 0.001	0.01 ± 0.001	0.01 ± 0.001	0.003 ± 0.001	0.004 ± 0.001	0.003 ± 0.001	>0.001
		Amies	16.0 ± 2.0	2.47 ± 0.3	3.98 ± 0.7	1.98 ± 0.4	0.22 ± 0.06	0.22 ± 0.04	0.003 ± 0.001
		Stuart	0.15 ± 0.02	0.01 ± 0.002	0.06 ± 0.01	>0.001	0.1 ± 0.01	0.005 ± 0.001	0.01 ± 0.002
		Dry	0.06 ± 0.003	0.01 ± 0.003	0.002 ± 0.001	>0.001	>0.001	>0.001	>0.001

*Yersinia pestis*	−70°C	PBST	1.93 ± 0.8	0.1 ± 0.02	0.03 ± 0.004	0.06 ± 0.005	0.23 ± 0.03	0.07 ± 0.01	0.1 ± 0.01
		Amies	2.13 ± 0.6	3.0 ± 0.2	1.4 ± 0.2	3.9 ± 0.3	2.8 ± 0.3	0.5 ± 0.04	1.7 ± 0.08
		Stuart	2.1 ± 0.6	6.3 ± 1.1	3.1 ± 0.5	2.8 ± 0.6	4.7 ± 0.4	2.2 ± 0.8	2.4 ± 0.2
		Dry	0.06 ± 0.01	0.4 ± 0.02	0.3 ± 0.05	0.6 ± 0.05	0.4 ± 0.03	0.3 ± 0.06	0.5 ± 0.01
	4°C	PBST	20.2 ± 2.2	7.8 ± 0.7	13.4 ± 1.8	4.8 ± 0.4	6.7 ± 0.5	3.8 ± 0.3	2.6 ± 0.2
		Amies	49.5 ± 3.6	37.8 ± 2.5	54.2 ± 3.2	53.6 ± 4.6	50.8 ± 4.8	12.8 ± 1.2	10.0 ± 1.1
		Stuart	41.3 ± 5.1	22.8 ± 1.2	32.8 ± 3.3	66.0 ± 6.2	51.8 ± 4.4	15.2 ± 1.3	32.0 ± 2.2
		Dry	12.0 ± 1.1	4.1 ± 0.4	0.08 ± 0.02	0.02 ± 0.005	>0.001	>0.001	>0.001
	25°C	PBST	6.6 ± 0.8	4.7 ± 0.3	3.8 ± 0.5	1.5 ± 0.1	0.7 ± 0.05	0.2 ± 0.03	0.2 ± 0.009
		Amies	52.3 ± 9.6	30.3 ± 1.4	34.2 ± 4.0	0.2 ± 0.002	2.1 ± 0.1	0.1 ± 0.03	0.005 ± 0.001
		Stuart	44.4 ± 9.8	49.0 ± 10.2	34.5 ± 5.2	46.6 ± 11.8	8.0 ± 1.4	0.04 ± 0.006	>0.001
		Dry	4.3 ± 0.5	0.6 ± 0.06	0.009 ± 0.002	0.0	0.0	−	−
	45°C	PBST	0.0	0.0	−	−	−	−	−
		Amies	1.3 ± 0.1	0.0	−	−	−	−	−
		Stuart	1.1 ± 0.1	>0.001	0.0	0.0	−	−	−
		Dry	0.0	0.0	−	−	−	−	−

VEE	−70°C	PBST			0.0	0.0	0.0		
		UTM			5.7 ± 1.1	5.48 ± 3.0	4.28 ± 0.23		
		M4RT			4.7 ± 0.8	10.9 ± 3.9	14.6 ± 2.8		
		Dry			0.13 ± 0.1	0.05 ± 0.001	0.09 ± 0.03		
	4°C	PBST			0.0	0.0	0.0		
		UTM			9.1 ± 1.3	0.8 ± 0.2	5.82 ± 2.4		
		M4RT			4.3 ±0.4	5.75 ± 0.23	9.45 ± 0.6		
		Dry			0.0	0.0	0.0		
	30°C	PBST			0.0	0.0	0.0		
		UTM			11.6 ± 9.9	0.0	0.0		
		M4RT			0.0	0.0	0.0		
		Dry			0.0	0.0	0.0		

**Table 2:** ECL immunodetection of *B. anthracis*, *Y. pestis,* and VEE. A four plus system was used to indicate the number of tests that were positive according to ECL immunodetection. Duplicate swabs were extracted at each time point and set up into duplicate detection assays. Thus there is a total of four luminescent readings to assess whether *B. anthracis*, *Y. pestis* and VEE displayed positive (+) or negative (−) detection. As in Table 1, in an effort to consolidate the large number of samples being extracted, VEE collection did not start until day 7 and persisted only until day 21. Table entries where there is discordance between bacterial and viral collection points have been grayed out.

Biothreat agent	Storage temp.	Storage media	Day 1	Day 7	Day 14	Day 21	Day 28	Day 60
*Bacillus anthracis*	−70°C	PBST	++++	++++	++++	++++	++++	++++
		Amies	++++	++++	++++	++++	++++	++++
		Stuart	++++	++++	++++	++++	++++	++++
		Dry	++++	++++	++++	++++	++++	++++
	4°C	PBST	++++	−	−	−	−	−
		Amies	++++	−	−	−	−	−
		Stuart	++++	−	−	−	−	−
		Dry	++++	++++	++++	++++	++++	++++
	25°C	PBST	++++	−	−	−	−	−
		Amies	−	−	−	−	−	−
		Stuart	++++	−	−	−	−	−
		Dry	−	−	−	−	−	−
	45°C	PBST	−	−	−	−	−	−
		Amies	−	−	−	−	−	−
		Stuart	−	−	−	−	−	−
		Dry	−	−	−	−	−	−

*Yersinia pestis*	−70°C	PBST	++++	++++	++++	++++	++++	++++
		Amies	++++	++++	++++	++++	++++	++++
		Stuart	++++	++++	++++	++++	++++	++++
		Dry	++++	++++	++++	++++	++++	++++
	4°C	PBST	++++	++++	++++	++++	++++	++++
		Amies	++++	++++	++++	++++	++++	++++
		Stuart	++++	++++	++++	++++	++++	++++
		Dry	++++	++++	++++	++++	++++	++++
	25°C	PBST	++++	++++	++++	++++	++++	++++
		Amies	++++	++++	++++	++++	++++	++++
		Stuart	++++	++++	++++	++++	++++	++++
		Dry	++++	++++	++++	++++	++++	++++
	45°C	PBST	++++	++++	++++	++++	++++	++++
		Amies	++++	++++	++++	++++	++++	++++
		Stuart	++++	++++	++++	++++	++++	++++
		Dry	++++	++++	++++	++++	++++	++++

VEE	−70°C	PBST		++++	++++	++++		
		UTM		++++	++++	++++		
		M4RT		++++	++++	++++		
		Dry		++++	++++	++++		
	4°C	PBST		++++	++++	++++		
		UTM		++++	++++	++++		
		M4RT		++++	++++	++++		
		Dry		++++	++++	++++		
	30°C	PBST		++++	++++	++++		
		UTM		++++	++++	++++		
		M4RT		++++	++++	++++		
		Dry		++++	++++	++++		

**Table 3:** ECL immunodetection of ricin toxin. A four plus system was used to indicate the number of tests that were positive according to ECL immunodetection. Duplicate swabs were extracted at each time point and set up into duplicate detection assays. Thus there is a total of four luminescent readings by which to assess whether a sample is positive (+) or negative (−) for ricin detection. Extraction and processing error on day 28 is indicated by NT (no data taken).

Biothreat agent	Storage temp.	Storage Media	Days in storage						
			1	3	7	14	21	28	60
Ricin high spike	−70°C	Water	++++	++++	++++	++++	++++	++++	++++
		PTM	++++	++++	++++	++++	++++	++++	++++
		Dry	++++	++++	++++	++++	++++	++++	++++
		PBST	++++	++++	++++	++++	++++	++++	++++
	4°C	Water	++++	++++	++++	++++	++++	++++	++++
		PTM	++++	++++	++++	++++	++++	++++	++++
		Dry	++++	++++	++++	++++	++++	++++	++++
	25°C	Water	++++	++++	++++	++++	++++	++++	++++
		PTM	++++	++++	++++	++++	++++	++++	++++
		Dry	++++	++++	++++	++++	++++	++++	++++
	45°C	Water	++++	++++	++++	++++	++++	++++	++++
		PTM	++++	++++	++++	++++	++++	++++	++++
		Dry	++++	++++	++++	+++	−	−	++++

Ricin low spike	−70°C	Water	++++	++++	++++	++++	++++	++++	++++
		PTM	++++	++++	++++	++++	++++	++++	++++
		Dry	++++	++++	++++	++++	++++	++++	++++
	4°C	Water	++++	++++	++++	++++	++++	++++	++++
		PTM	++++	++++	++++	++++	++++	++++	++++
		Dry	++++	++	+	+	−	++++	++++
	25°C	Water	++++	++++	++++	+	−	−	++++
		PTM	++++	++++	−	++	−	−	++++
		Dry	−	−	−	−	−	NT	++
	45°C	Water	−	−	−	−	−	NT	−
		PTM	−	−	−	−	−	NT	++++
		Dry	−	−	−	−	−	NT	−
