# Supplementary material for: Suitability of Commercial Transport Media for Biological Pathogens under Nonideal Conditions
Source: Int J Microbiol. 2011 Oct 30;2011:463096. doi: 10.1155/2011/463096 (PMC3206327; doi:10.1155/2011/463096)
Supplement: Supplementary file 1 — Supplemental Figure 1 depicts microscopic images, captured using a 100X objective, ofB. anthracis sporulation in PBST, liquid Amies and liquid Stuart transport media on day seven. Vegetative cells are seen as long filaments in the stock culture on day zero in PBST (panels on the far left). Spores are observed as small refractive bodies either within the filamentous cells (or cell fragment) or free within the transport media (panels on the right). Supplemental Figures 2–4 demonstrate nucleic acid detection using real-time PCR for Y. pestis, B. anthracis and VEE respectively. Each figure summarizes the real-time detection data for each transport media at each storage temperature: −70°C (A), 4°C (B), 25°C (C) and 45°C (D) for Y. pestis and B. anthracis, −70°C (A), 4°C (B) and 30°C (C) for VEE. Data points represent the average Ct value for two PCR reactions over the 60-day test period. [file 463096.f1.pdf]

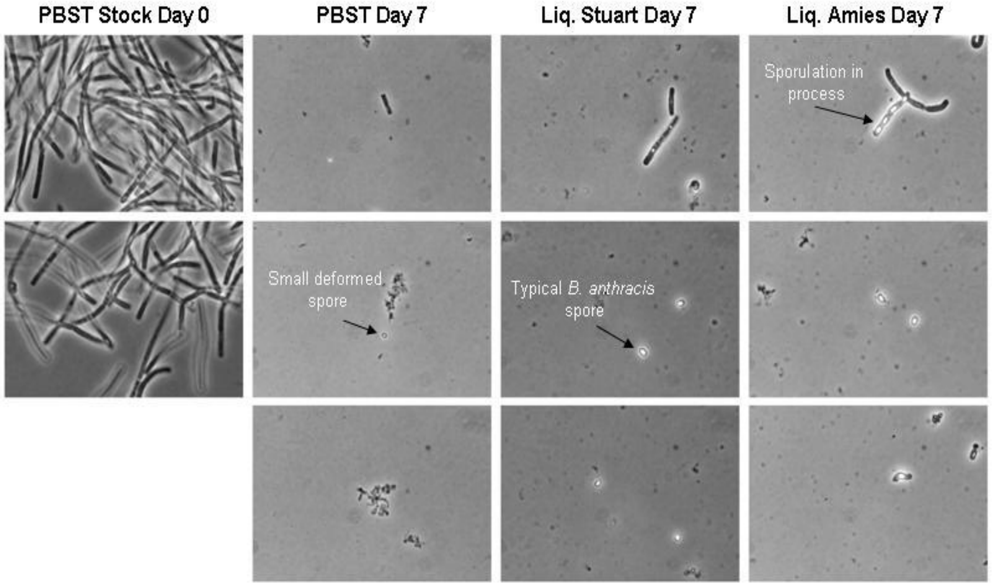

Supplemental Figure 1. Sporulation of *B. anthracis* Sterne in transport media. Microscopic digital images captured using 100× objective. Vegetative cells are seen as long filaments in the stock culture on day zero (panels on the far left). Spores are observed as small refractive bodies either within the filamentous cells (or cell fragment) or free within the transport media (panels on the right).

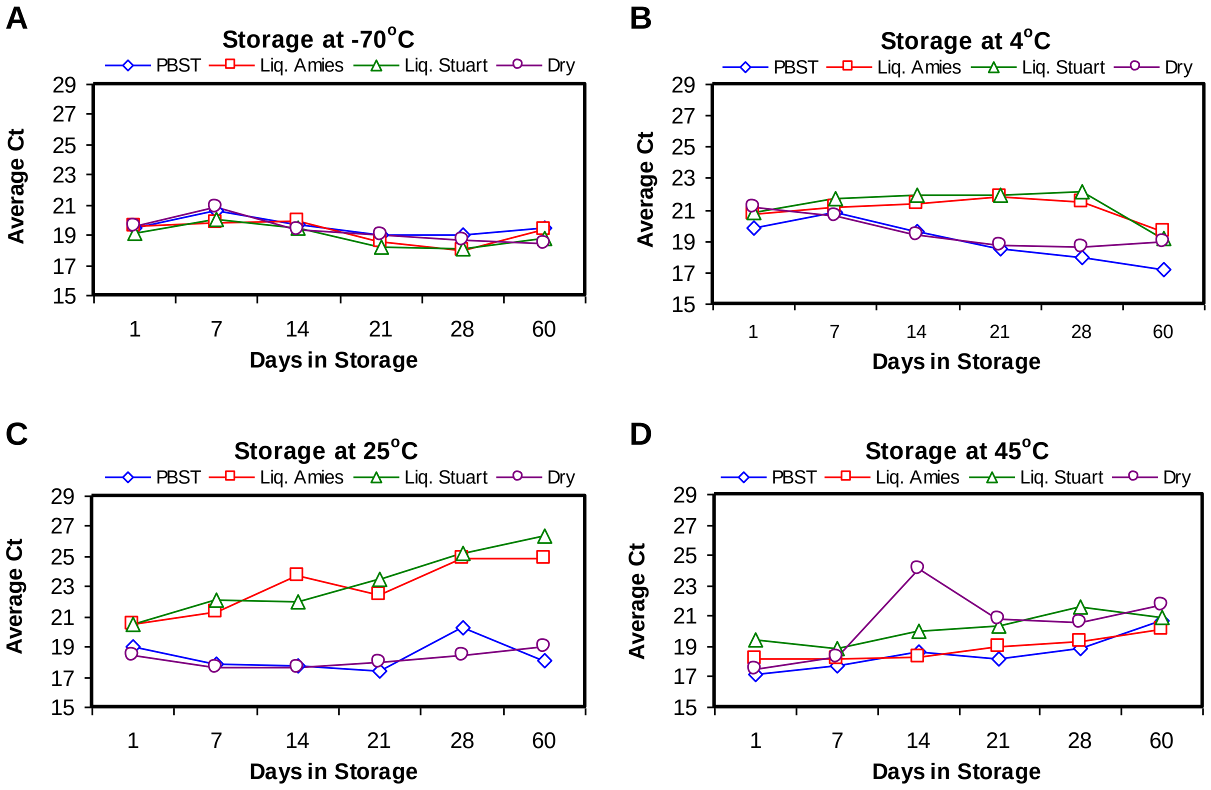

Supplemental Figure 2. Detection of *Y. pestis* A1122 nucleic acid signature using real-time PCR. Each graph summarizes the real-time detection data for each storage temperature -70°C (A), 4°C (B), 25°C (C) and 45°C (D). Data points represent the average C_t value for two PCR reactions.

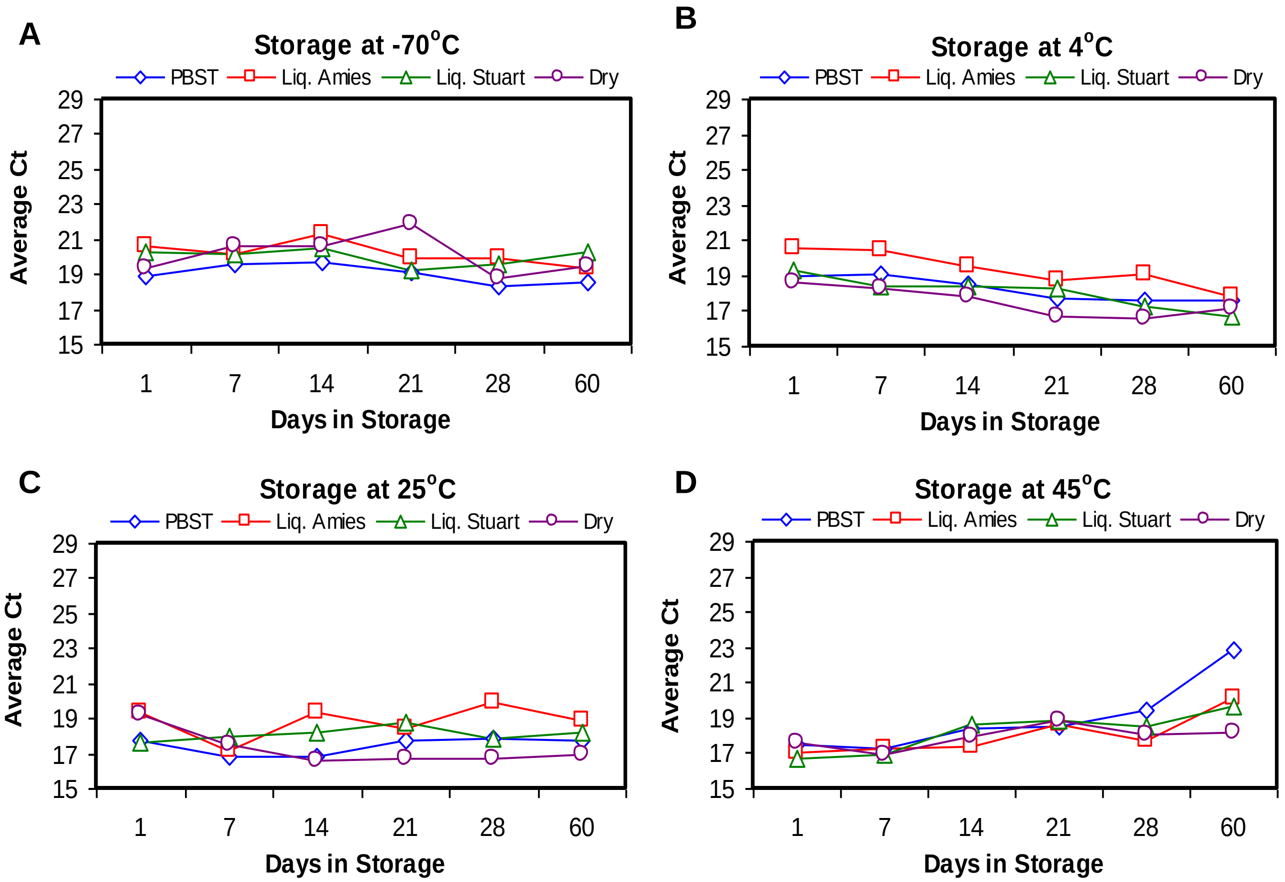

Supplemental Figure 3. Detection of *B. anthracis* Sterne nucleic acid signature using real-time PCR. Each graph summarizes the real-time detection data for each storage temperature. Data points represent the average C_t value for two PCR reactions.

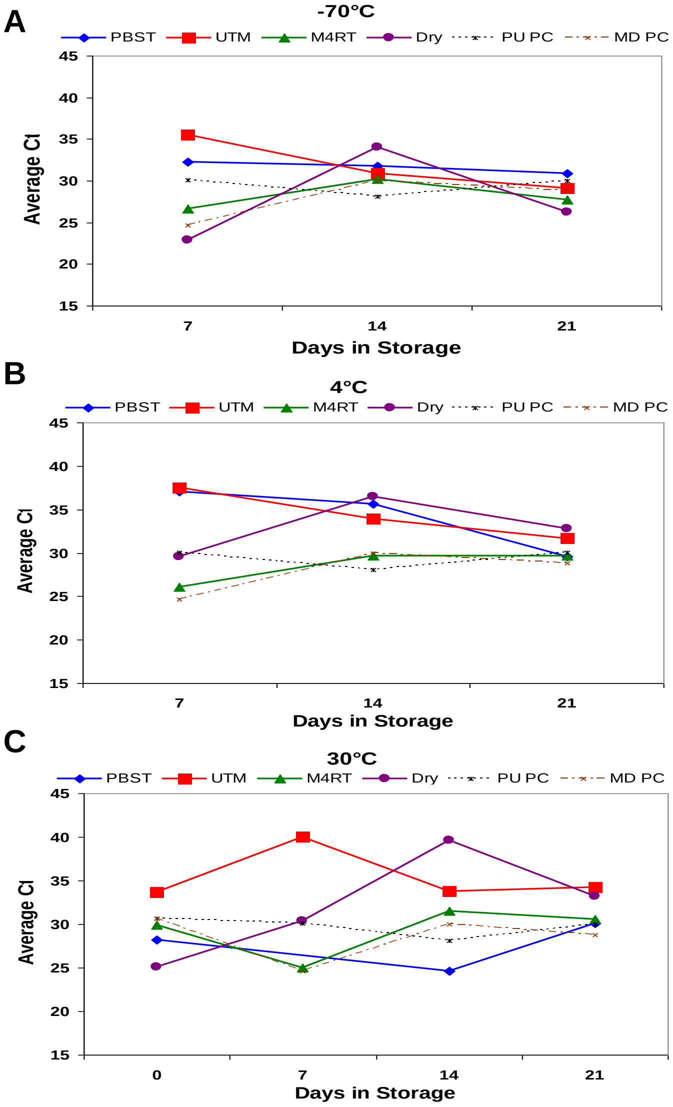

Supplemental Figure 4. Detection of VEE virus nucleic acid signature using real-time PCR. Each graph summarizes the real-time detection data at (A) -70°C, (B) 4°C and (C) 30°C. Data points represent the average Ct value for two PCR reactions. There were two separate PCR assays per time point. Positive controls for each assay are indicated by the dashed lines (PU PC = PBST/UTM positive control and MD PC = M4RT/Dry positive control).
